# The solution structure of the prototype foamy virus RNase H domain indicates an important role of the basic loop in substrate binding

**DOI:** 10.1186/1742-4690-9-73

**Published:** 2012-09-10

**Authors:** Berit Leo, Kristian Schweimer, Paul Rösch, Maximilian J Hartl, Birgitta M Wöhrl

**Affiliations:** 1 Universität Bayreuth, Lehrstuhl Biopolymere, Universitätsstr. 30, D-95447, Bayreuth, Germany

**Keywords:** Foamy virus, Retroviral RNase H, C-helix, Basic loop, NMR, Basic protrusion, Solution structure, Substrate binding

## Abstract

**Background:**

The ribonuclease H (RNase H) domains of retroviral reverse transcriptases play an essential role in the replication cycle of retroviruses. During reverse transcription of the viral genomic RNA, an RNA/DNA hybrid is created whose RNA strand needs to be hydrolyzed by the RNase H to enable synthesis of the second DNA strand by the DNA polymerase function of the reverse transcriptase. Here, we report the solution structure of the separately purified RNase H domain from prototype foamy virus (PFV) revealing the so-called C-helix and the adjacent basic loop, which both were suggested to be important in substrate binding and activity.

**Results:**

The solution structure of PFV RNase H shows that it contains a mixed five-stranded β-sheet, which is sandwiched by four α-helices (A-D), including the C-helix, on one side and one α-helix (helix E) on the opposite side. NMR titration experiments demonstrate that upon substrate addition signal changes can be detected predominantly in the basic loop as well as in the C-helix. All these regions are oriented towards the bound substrate. In addition, signal intensities corresponding to residues in the B-helix and the active site decrease, while only minor or no changes of the overall structure of the RNase H are detectable upon substrate binding. Dynamic studies confirm the monomeric state of the RNase H domain. Structure comparisons with HIV-1 RNase H, which lacks the basic protrusion, indicate that the basic loop is relevant for substrate interaction, while the C-helix appears to fulfill mainly structural functions, i.e. positioning the basic loop in the correct orientation for substrate binding.

**Conclusions:**

The structural data of PFV RNase H demonstrate the importance of the basic loop, which contains four positively charged lysines, in substrate binding and the function of the C-helix in positioning of the loop. In the dimeric full length HIV-1 RT, the function of the basic loop is carried out by a different loop, which also harbors basic residues, derived from the connection domain of the p66 subunit. Our results suggest that RNases H which are also active as separate domains might need a functional basic loop for proper substrate binding.

## Background

RTs of retroviruses are multifunctional enzymes, which in addition to a polymerase domain harbor a C-terminal RNase H domain whose activity is essential during reverse transcription. The RNase H function is necessary for degrading the RNA in the emerging RNA/DNA hybrid [1] to allow synthesis of the second DNA strand. In contrast to orthoretroviruses, spumaretroviruses, also called foamy viruses (FVs), possess a mature protease-reverse transcriptase (PR-RT) in the virion, whereas all other retroviruses cleave off the PR domain from the Pol precursor [2,3]. Similarly to the RT from Moloney murine leukemia virus (MoMLV) [4], the PR-RT of the prototype foamy virus (PFV) is also monomeric [3].

We have shown previously, that the isolated RNase H domain of PFV exhibits activity, albeit to a much lower extent than the mature full length PR-RT [5]. This behavior is comparable to that of the separate RNase H domain of MoMLV [6-8]. Furthermore, NMR spectroscopy of PFV RNase H revealed the existence of the so-called “basic protrusion” which consists of an α-helix, referred to as the “C-helix” followed by a basic loop [5]. The basic protrusion of RNases H has been implicated in substrate binding and activity [9-11] and has also been found in the retroviral RNase H domain of MoMLV [12] and in the recently solved x-ray structure of xenotropic murine leukemia virus-related virus (XMRV) [13]. XMRV is highly related to MoMLV and appears to have arisen through recombination events during passaging of human tumors in mice [14].

A basic protrusion can also be found in the human and *Escherichia coli* (*E. coli*) RNases H, whereas the RNase H from *Bacillus halodurans* (*B. halodurans*) contains only the basic loop but no C-helix. However, an N-terminal RNA/DNA-hybrid binding domain (RHBD) compensates for the lack of the helix [15]. In contrast, HIV-1 RNase H, which is not active when expressed in isolation [16-20], does not harbor the basic protrusion, rather, positively charged residues in the connection domain assume this function.

Here, we show the solution structure of PFV RNase H and titration experiments with an RNA/DNA substrate indicating an important role of the basic loop in substrate binding.

## Results and discussion

To determine the solution structure of the wild type (wt) PFV RNase H we purified the recombinant ^15^ N or ^15^ N, ^13^C labeled protein PFV RNase H-(Q^591-^N^751^) from *E. coli*. We have shown previously, that the purified domain exhibits RNase H activity and possesses a defined tertiary structure including the C-helix [5].

### Structure of the isolated PFV RNase H

To verify the oligomerization state of the PFV RNase H domain ^15^ N spin relaxation experiments were performed. Comparison of ^15^ N transverse (R_2_) and longitudinal (R_1_) relaxation rates at two different concentrations (1 mM and 0.4 mM) reveals deviations of less than 6 % for R_2_ and 3 % for R_1_, indicating that no aggregation is observed under the conditions applied for NMR spectroscopy. From the relaxation rates, a rotational correlation time (tc) of 12.4 ns was determined for an isotropic model. In addition, the data were fitted to an axial symmetric as well as a fully asymmetric rotational diffusion tensor using the coordinates from the lowest energy structure, but no further statistical improvement was obtained. The higher tc of PFV RNase H as compared to the isolated RNase H domain of HIV-RT (9.7 ns, [21]) reflects the larger size of the PFV RNase H (165 aa vs. 138 aa, 18 kDa vs. 15.3 kDa), and confirms the monomeric character of the domain already demonstrated by size exclusion chromatography [5].

**Figure 1:**
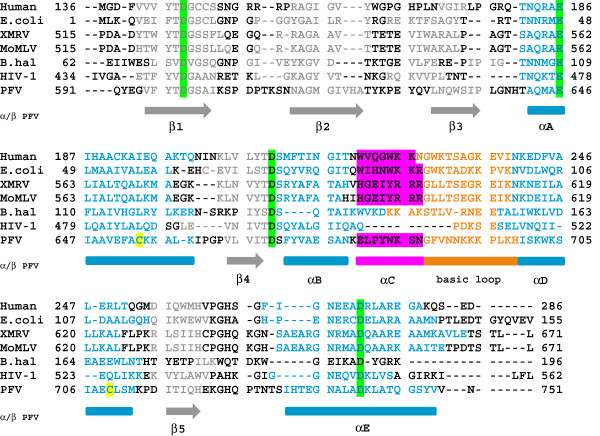
**Sequence alignment of different RNase H domains.** The RNases H from human origin, *E. coli,* XMRV, MoMLV, *B. haludorans,* HIV-1 and PFV are shown. The numbers represent the amino acid numbers of the corresponding full length enzymes. The alignment was performed with the program CLC Protein Workbench 5 (version 5.3). The arrows and boxes below indicate the secondary structures determined for PFV RNase H; α-helices are shown in blue, β-strands in gray. The active site residues are highlighted in bright green; the cysteines (C654, C709) of PFV RNase H are marked in yellow. The C-helices and basic loops of the enzymes are highlighted in magenta and orange, respectively.

**Figure 2:**
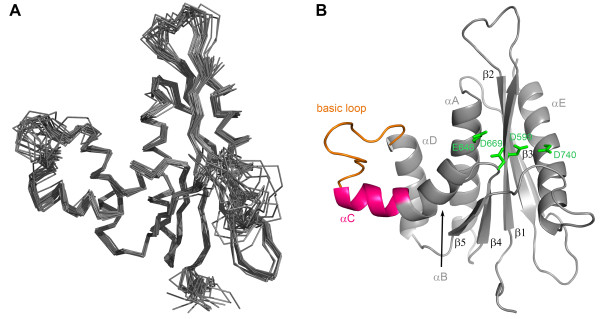
**Solution structure of PFV RNase H. **(**A**) Superposition of the 19 lowest energy structures. (**B**) Ribbon diagram of the lowest energy structure. The C-helix is highlighted in magenta, and the basic loop in orange. The active site residues D599, E646, D669 and D740 are highlighted in bright green.

Sequence comparisons (Figure 1) indicated that similar to MoMLV and XMRV RNase H, PFV RNase H contains a basic protrusion, including the so-called C-helix followed by a basic loop structure. We have shown previously that the purified, separate PFV RNase H domain is weakly active [5]. Structural analyses by NMR spectroscopy revealed the presence of a structured C-helix [5]. Thus, we set out to determine the solution structure of PFV RNase H (Figure 2). 

Using multidimensional heteronuclear edited NOESY experiments, 1686 distance restraints together with 66 restraints for hydrogen bonds and 225 dihedral angle restraints based on chemical shift analyses could be derived (Table 1). The final structure calculation resulted in an ensemble of structures with no distance restraint violation larger than 0.1 Å and no dihedral restraint violation larger than 2.4° together with good stereochemical properties (Table 1). The structures superimpose well in the secondary structure regions (Figure 2A). For several loop regions no distance restraints could be derived because of unassigned residues [5]. Their NMR signals are most likely broadened beyond detection due to exchange processes. Therefore the large coordinate variability reflects the dynamic character of these regions. 

**Table 1:** Solution structure statistics

**Experimentally derived restraints**		
distance restraints		
	NOE	1686
	intraresidual	527
	sequential	343
	medium range	245
	long range	505
	ambiguous	66
	hydrogen bonds	62
dihedral restraints		225
**Restraint violation**		
average distance restraint violation (Å)	0.0035 +/− 0.0008	
maximum distance restraint violation (Å)	< 0.1	
average dihedral restraint violation (°)	0.10 +/− 0.04	
maximum dihedral restraint violation (°)	2.4	
**deviation from ideal geometry**		
bond length (Å)	0.00064 +/− 0.00005	
bond angle (Å)	0.12 +/− 0.006	
**Coordinate precision**^**a,b**^		
backbone heavy atoms (Rmsd) (Å) (all / defined secondary structure )	1.05 / 0.46	
all heavy atoms (Rmsd) (Å)	1.58 / 0.92	
**Ramachandran plot statistics**^**c**^ (%)	89.7/9.6/0.3/0.4	

^a^ The precision of the coordinates is defined as the average atomic root mean square difference between the accepted simulated annealing structures and the corresponding mean structure calculated for the given sequence regions.

^b^ calculated for residues 593–747 (all) or 594–604, 612–621, 629–637, 642–659, 664–668, 671–686, 700–712, 717–720, 731–747 (defined secondary structure).

^c^ Ramachandran plot statistics are determined by PROCHECK and noted by most favored/additionally allowed/generously allowed/disallowed.

The structure of PFV RNase H shows the typical tertiary fold of an RNase H domain, which is formed by a five-stranded mixed β-sheet flanked by five α-helices. The long carboxy-terminal helix E packs on one side of the β-sheet, and the helices A, B, C and D are located on the other side of the β-sheet (Figure 2B). Due to a lack of structural restraints accessible by NMR, no attempts were made to characterize the active site. Despite the absence of certain restraints in this region, the active site residues (D599, E646, D669, D740) are closely enough together to allow coordination of magnesium ions.

A characteristic feature of PFV RNase H is the presence of helix C which directly follows helix B after a kink. Helix C precedes a basic loop containing three neighboring lysines. Helix C as well as the adjacent loop is also found in XMRV RNase H. However, in XMRV RNase H the consecutive basic residues (3 x Arg) are part of helix C [13], whereas in PFV RNase H four lysines (**KKK**PL**K**) are located in the adjacent basic loop (Figure 1). The slightly different position of the basic residues might cause different distances to the active site and might thus contribute to possible differences in cleavage activities of the two enzymes [5,13,22]. The orientation of helix C is determined by numerous hydrophobic contacts to helix D. This suggests that helix C acts as a molecular ruler to orient the basic loop in order to optimize substrate binding and specificity.

**Figure 3:**
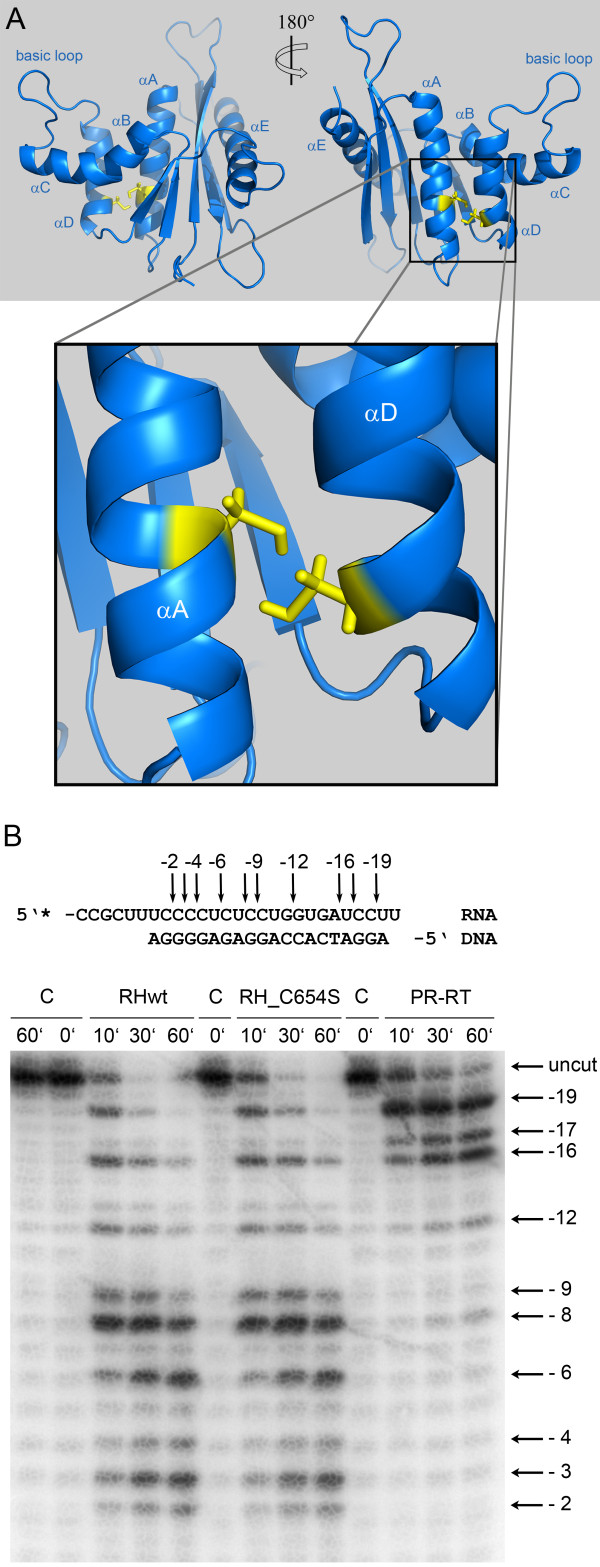
**Orientation of Cys654 and Cys709 in PFV RNase H. **(**A**) Enlargement of α-helices A and D of PFV RNase H*.* The blow-up shows the structural elements αA and αD, harboring residues C654 and C709, respectively*.* (**B**) Qualitative RNase H activity assay*.* Comparison of RNase H mutant C654S (RH_C654S) with the wt RNase H (RHwt) and the full length PR-RT. Reaction products were separated on a denaturing sequencing gel and visualized by phosphoimaging. The 27/20-mer RNA/DNA substrate, ^32^P-labeled at the 5’ end of the RNA, is shown on top of the gel. Arrows and numbers indicate the cleavage sites identified on the sequencing gel.

The structure of PFV RNase H further revealed that two cysteine residues, Cys654 and Cys709 (Figure 1), located in helix A and D, respectively, are facing each other, thus allowing the potential formation of a disulfide bridge (Figure 3A). To find out whether this disulfide bridge is important for RNase H function, we mutated Cys654 to serine to abolish disulfide formation. However, analyses of the RNase H activities of the mutant RNase H-(C654S) and the wt enzyme revealed no differences in activity (Figure 3B), indicating that creation of the disulfide bridge is not relevant for RNase H activity. Sequence comparisons of PFV RNase H with other foamy virus RNases H (feline, bovine) showed, that the cysteines are not conserved. Only the closely related RNase H from simian FV from macaques (SFVmac) retains the cysteines (data not shown). Other RNases H also do not contain two cysteines facing each other (Figure 1).

### Substrate binding

**Figure 4:**
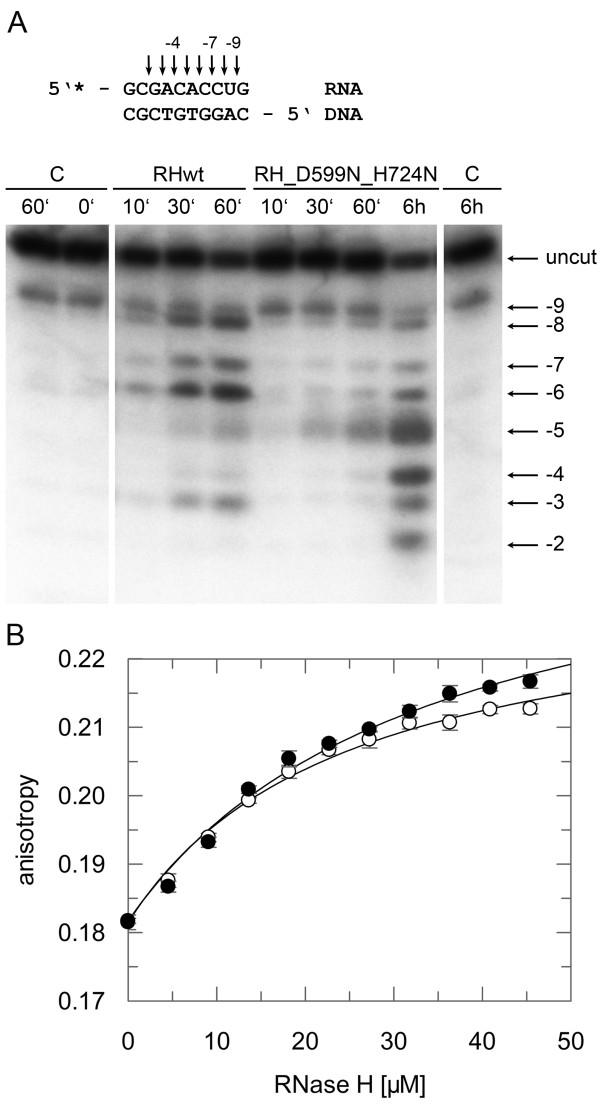
**RNase H activity and binding affinities of mutant RNase H (D599N-H724N). **(**A**) Qualitative RNase H assay. The 10/10-mer RNA/DNA substrate, 5’ labeled at the RNA strand with ^32^P, is depicted on top of the figure. Cleavage products obtained with 670 nM substrate and 20 μM wt RNase H (RHwt) or mutant RNase H-(D599N-H724N) (RH_D599N_H724N) were separated on a 15 % sequencing gel and visualized by phosphoimaging. Arrows and numbers indicate the cleavage sites identified on the gel. The first nucleotide of the RNA hybridized to the 3’ OH nucleotide of the DNA strand is designated −1. The incubation times are shown on top of the gel. C, control assays were performed in the absence of enzyme for 0 minute, 60 minutes and 6 hours. (**B**) Determination of K_D_-values by fluorescence anisotropy measurements. 25 nM of the 10/10-mer RNA/5’-DY647-DNA substrate were titrated with increasing amounts of wt RNase H (open circles) or mutant RNase H-(D599N-H724N) (closed circles). The fit of the curve to the data [5] resulted in K_D_-values of 23 μM (± 3) for the wt, and 31 μM (± 5) for the mutant RNase H-(D599N-H724N) enzyme.

To analyze the interaction of PFV RNase H with an RNA/DNA substrate, we had to abolish RNase H activity to avoid cleavage of the substrate during long-term NMR analyses. Thus, we mutated the active site residue Asp599 and, in addition, residue His724, located in the loop region between β-strand 5 and helix E to asparagine. The corresponding histidine residue in HIV-1 RNase H had been shown to severely reduce RNase H activity [23,24]. The RNase H activity of the double mutant D599N-H742N was then analyzed qualitatively with the same 10/10-mer RNA/DNA substrate, which we wanted to use further in NMR analyses. Our results demonstrate that the RNase H activity of the mutant is seriously impaired (Figure 4). However, only minor changes were observed in the recorded NMR spectra, indicating that the overall tertiary structure of the RNase H domain is unaffected by the amino acid exchanges (data not shown). 

Furthermore, we introduced the two amino acid exchanges into the full length PR-RT to determine the impact of the mutations in DNA polymerization. Analysis of the polymerization activities on a homopolymeric substrate resulted in similar specific activities of 8.2 U and 8.8 U (per μg of enzyme) for the wt and mutant PR-RT-(D599N-H742N), respectively, revealing that the mutations do not impair polymerase activity and overall structure of PFV PR-RT.

To determine the binding affinity of the mutant RNase H-(D599N-H742N) we used the same 10/10-mer substrate, now labeled with the fluorescence marker DY647 to allow fluorescence anisotropy measurements [5]. Despite of its low RNase H cleavage activity, the mutant revealed comparable binding affinities for the RNA/DNA substrate (K_D_ ≈ 31 μM ± 5) as the wt enzyme (K_D_ ≈ 23 μM ± 3) (Figure 4B). This characteristic made the double mutant D599N-H742N suitable for NMR titration experiments.

### NMR titration experiments

**Figure 5:**
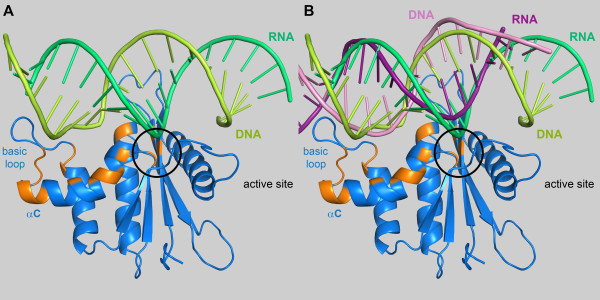
**Modeling of an RNA/DNA substrate onto PFV RNase H.** Ribbon diagram of mutant RNase H-(D599N-H724N). The regions highlighted in orange exhibited significant signal changes upon substrate addition. (**A**) Based on the structure of the human RNase H/substrate complex (PDB: 2QK9) a structural alignment of the PFV and human RNase H was performed to model the RNA/DNA substrate of the human RNase H onto PFV RNase H (Rmsd: 2.35 Å). The RNA strand is shown in dark green, the DNA strand in light green. (**B**) Alignment of the complex shown in (**A**) with the HIV-1 RT/RNA-DNA complex (PDB: 1HYS) harboring the PPT RNA-DNA. The PPT substrate is depicted in dark (RNA) and light purple (DNA). The location of the active site residues of PFV RNase H is indicated by a black circle.

^1^ H-^15^ N-HSQC spectra of 50 μM mutant PFV RNase H-(D599N-H742N) were recorded using increasing molar ratios of RNA/DNA:protein of 0, 1, 2, 3, 4 and 5. Upon substrate addition a decrease of the signal intensities of several residues (G600, A614, T641, Q643, D669, F671, A674, E675, S676, E680, K685, S686, N687, F689, V690, N691, H699, I700) could be observed. A signal change was considered relevant, if the signal decreased to 50 % or less of the original value. The residues potentially involved in substrate binding are highlighted in orange in Figure 5. Apart from the active site residues, helix B and C as well as the basic loop appear to be involved in substrate binding. The similarity of PFV RNase H and human RNase H allowed the generation of a simple model of PFV RNase H in complex with substrate. Thus, we superimposed the PFV RNase H structure with that of the human RNase H/substrate complex (PDB: 2QK9) to determine whether the residues, which show significant effects in the NMR titration experiments, are located spatially close to the substrate (Figure 5A). The alignment indicates a potential role of helix C in positioning the basic loop close to the DNA strand, thus supporting substrate binding, whereas the active site residues are close to the RNA strand, which has to be cleaved during catalysis.

Further information on substrate binding is provided by the alignment of the complex shown in Figure 5A with HIV-1 RT (PDB: 1HYS) in complex with a polypurine tract (PPT) containing RNA/DNA hybrid [25]. Only the PPT hybrid substrate modeled onto the PFV RNase H is shown (Figure 5B). The modeling reveals that the PPT RNA of the hybrid is not as close to the active site as the RNA strand from the other substrate depicted in Figure 5A. This is not surprising since the PPT-RNA/DNA hybrid, which is not cleaved by the RNase H during reverse transcription, adopts a peculiar structure in the complex with HIV-1 RT [25]. In the PPT region, which is rich in adenines, an unpaired base on the DNA strand takes the base pairing out of register leading to two offset base pairs. Then, an unpaired base on the RNA strand re-establishes the normal register. This structural deviation of PPT containing substrates was suggested to play a role in the resistance of the PPT to RNase H cleavage [25].

A model of XMRV RNase H complexed with substrate, published recently, suggests that several aromatic residues (Y586, H594 and Y598) located in helices B and C are capable of hydrogen-bond or stacking interactions with the substrate [13]. The corresponding residues in PFV RNase H are Y672 (located in helix B), K679 (located between helix B and C) and Y683 (located in helix C). Since these residues are solvent exposed in PFV RNase H, substrate interaction might also be possible. These results suggest that monomeric RNase H domains that are active in isolation might need the basic protrusion to confer sufficient affinity for the substrate to permit cleavage.

**Figure 6:**
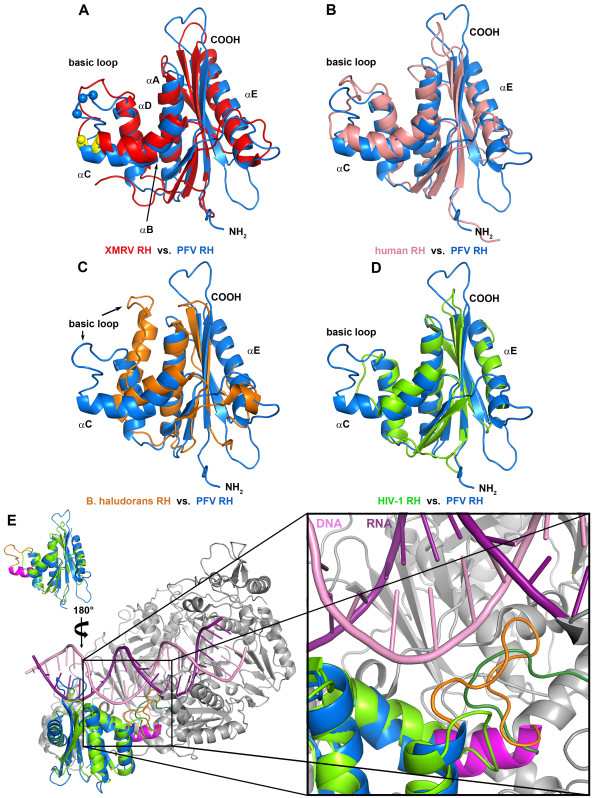
**Structural overlay of PFV RNase H with different RNase H structures and HIV-1 RT. **(**A – D**) Superposition of the lowest energy structure of PFV RNase H with different RNases H*.* (**A**) XMRV (PDB: 3V1O, Rmsd: 2.10 Å), the C-α positions of the three positively charged arginine (XMRV) or lysine (PFV) residues in the basic protrusion are highlighted as yellow (XMRV) or blue (PFV) spheres, (**B**) human (PDB: 2QK9, Rmsd: 2.35 Å), (**C**) *B. halodurans* (PDB: 1ZBF, Rmsd: 2.37 Å), and (**D**) HIV-1 (PDB: 1HRH, Rmsd: 1.90 Å). (**E**) Orientation of PFV and HIV-1 RNase H in the full length HIV-1 RT. Superposition of the RNase H domains from PFV (blue) (PDB: 2LSN) and HIV-1 (green) (PDB: 1HYS) in the full length HIV-1 RT (gray) (PDB: 1HYS) in complex with a DNA/RNA substrate (Rmsd: 1.85 Å). With regard to (**D**) the RNase H domains are turned vertically by 180°C. The enlargement shows the region harboring the C-helix (magenta) and the basic loop (orange) of PFV RNase H and the corresponding loop (dark green) derived from the connection domain of the p66 subunit of the heterodimeric HIV-1 p66/p51 RT. The RNA strand of nucleic acid substrate is colored in dark purple, the DNA strand is in light purple.

To further analyze the structural features of PFV RNase H we performed structural comparisons with the RNases H of *B. halodurans*[15], human origin [26], XMRV [13] as well as HIV-1 [20] (Figure 6). The structure of PFV RNase H most closely resembles those of XMRV and HIV-1, followed by human RNase H, while the orientation of the basic loop in *B. halodurans* RNase H is strikingly different (Figure 6C). However, similar to HIV-1 RNase H (Figure 6D), *B. halodurans* RNase H also lacks the C-helix, but harbors an additional N-terminal extension instead, which was shown to be involved in substrate binding [15]. Superposition of PFV RNase H with HIV-1 RNase H (Figure 6D) demonstrates that due to the lack of the C-helix in HIV-1 the adjacent loop is too short and is not positioned correctly for substrate binding. 

To obtain a clearer picture of the orientation of these loops, we superimposed the structure of the full length HIV-1 RT in complex with the PPT containing hybrid (PDB: 1HYS) with PFV RNase H (Figure 6E). The enlargement of the region where the basic protrusion of PFV RNase H is located clearly shows that the basic loop is close to the substrate, whereas the corresponding loop of HIV-1 RNase H is much too short to contact the nucleic acid. Rather, a different loop derived from the connection domain (R356 – N363) of the p66 subunit of the heterodimeric HIV-1 RT appears to fulfill this function [27]. Similarly to the basic loop of PFV RNase H, which contains several lysine residues, this loop of HIV-1 RT also contains basic residues (R356, R358).

## Conclusions

In summary, we were able to identify regions in the RNase H domain - and by structural alignments also in the connection domain of HIV-1 RT - that interact with the substrate. Our data suggest that the basic loop plays an important role in substrate binding and that the major role of the C-helix is to orientate the basic loop properly towards the substrate. Furthermore, regions in the B-helix, which are also present in HIV-1 RNase H, have been identified to be involved in substrate binding. Together, this information might lead to a better understanding of the mechanism of action of HIV RNase H inhibitors and can contribute to the design and development of more specific and more potent inhibitors.

## Methods

### Mutant plasmid construction

The plasmid pET-GB1a-RNase H-(Q^591-^N^751^) coding for the gene of the wt PFV RNase H-(Q^591-^N^751^) as described previously [5] was used as a template for site-directed mutagenesis to obtain the mutant proteins RNase H-(C654S) and RNase H-(D599N-H724N) using the following primers:

(i) mutation C654S:

5’-GCTGCAGTTGAATTTGCCAGTAAAAAAGCTTTAAAAATACCTGG;

5’-CCAGGTATTTTTAAAGCTTTTTTACTGGCAAATTCAACTGCAGC;

(ii) mutation D599N:

5’-GAAGGAGTGTTTTATACTAATGGCTCGGCCATCAAAAGTCC

5’-GGACTTTTGATGGCCGAGCCATTAGTATAAAACACTCCTTC

(iii) mutation H724N:

5’-CTATTCAACATGAAAAAGGGAATCAGCCTACAAATACCAGTAT

5’-ATACTGGTATTTGTAGGCTGATTCCCTTTTTCATGTTGAATAG.

Point mutations were introduced according to the QuikChange kit (Stratagene, Waldbronn, Germany) and confirmed by sequencing (LGC Genomics, Berlin, Germany). Mutations coding for the amino acid exchanges D599N and H724N were introduced into the wt full length PR-RT using the same primers as for the mutation of the isolated RNase H domain.

### Gene expression and protein purification

Protein purifications of PFV RNase H wt, RNase H-(C654S) and RNase H-(D599N-H724N) were performed as described previously [5]. PFV PR-RT(D599N-H724N) as well as ^15^ N-labeled mutant RNase H-(D599N-H724N) and ^15^ N,^13^C labeled wt RNase H used in NMR studies were also purified as described previously [3,5].

### Qualitative RNase H assay

Reactions were performed as described previously [5] with 20 μM wt RNase H, RNase H-(C654S) and RNase H-(D599N-H724N), respectively, using a 10/10-mer or a 27/20-mer RNA/DNA substrate, whose RNA was 5’ end labeled with ^32^P [5]. The sequence of the 10-mer RNA was 5’-GCGACACCUG, the 10-mer DNA sequence was complementary to it. Sequences of the 27/20-mer RNA/DNA hybrid have been published previously [5]. After preincubation of the sample for 5 minutes at 25°C, reactions were started by the addition of ca. 670 nM 10/10-mer or 240 nM 27/20-mer RNA/DNA hybrid. Aliquots were taken as indicated in the figures and processed further as described previously [5].

### Fluorescence anisotropy measurements

Fluorescence equilibrium titrations were performed as described [5] to determine the dissociation constant (K_D_) of wt RNase H and mutant RNase H-(D599N-H724N) employing 25 nM of the 10/10-mer RNA/DNA substrate used above for the qualitative RNase H activity assay, however with the fluorescence label DY647 attached at the 5’ end of the DNA (DNA-5’-DY647-CAGGTGTCGC) (biomers.net GmbH, Ulm, Germany). The excitation wavelength was 652 nm and emission was detected at 673 nm. The slit widths were set at 8 nm and 7 nm for excitation and emission, respectively. The standard deviation for each data point is represented by error bars in Figure 4B. Calculation of the K_D_-values was performed as described previously [5].

### NMR analyses

NMR samples containing 0.4 – 1.0 mM uniformly ^15^ N or ^13^C, ^15^ N labeled RNase H were analyzed in 5 mM Na-phosphate, pH 7.0, 100 mM NaCl, 0.5 mM DTT, 10 % D_2_O (v/v), 6 mM MgCl_2_. All NMR experiments were conducted at 298 K on a Bruker Avance 600 MHz or a Bruker Avance 700 MHz (equipped with a cryogenic probe) spectrometer. In addition to the previously described experiments for resonance assignments [5]^15^ N- and ^13^C-edited NOESY experiments (mixing time 120 ms) were acquired for obtaining distance restraints. Standard ^15^ N spin relaxation experiments [28,29] were conducted at 600 MHz ^1^ H NMR frequency for obtaining transverse and longitudinal ^15^ N relaxation rates.

For NMR titration experiments a 1.2 fold molar excess of the non-labeled 10-mer DNA was hybridized to the complementary non-labeled 10-mer RNA in 50 mM Na-phosphate, pH 7.0, 100 mM NaCl by heating the sample to 95°C for 2 minutes, followed by transfer to a heating block at 70°C and slow cooling to room temperature. 50 μM ^15^ N-labeled mutant RNase H-(D599N-H724N) was dissolved in 50 mM Na-phosphate, pH 7.0, 100 mM NaCl, 6 mM MgCl_2_, 2 mM DTT, 10 % D_2_O (v/v). Different molar ratios of RNA/DNA:protein of 0, 1, 2, 3, 4 and 5 were employed.

### Structure calculation

Distance restraints for structure calculation were derived from ^15^ N-edited NOESY and ^13^C-edited NOESY spectra. NOESY cross peaks were classified according to their relative intensities and converted to distance restraints with upper limits of 3.0 Å (strong), 4.0 Å (medium), 5.0 Å (weak), and 6.0 Å (very weak). For ambiguous distance restraints the r^-6^ summation over all assigned possibilities defined the upper limit. Experimental NOESY spectra were validated semi-quantitatively against back-calculated spectra to confirm the assignment and to avoid bias of upper distance restraints by spin diffusion. Dihedral restraints were taken from analysis of chemical shifts by the TALOS software package [30]. Hydrogen bonds were included for backbone amide protons in regular secondary structure, when the amide proton did not show a water exchange cross peak in the ^15^ N-edited NOESY spectrum.

The structure calculations were performed with the program XPLOR-NIH 1.2.1 ([31]) using a three-step simulated annealing protocol with floating assignment of prochiral groups including a conformational database potential. The 19 structures (out of total 120 structures) showing the lowest values of the target function excluding the database potential were further analyzed with X-PLOR, PROCHECK [32] and pyMOL [33].

### Polymerization assay

RNA-dependent DNA polymerase activities of PFV PR-RT and PFV PR-RT(D599N-H724N) were quantitated at 37°C on a poly(rA)/oligo(dT)_15_ substrate (0.2 U/ml) (Roche Diagnostics GmbH, Mannheim, Germany) in a reaction volume of 24 μl and 50 mM Tris/HCl, pH 8.0, 80 mM KCl, 6 mM MgCl_2_, 0.5 mM DTT with 150 μM unlabeled TTP and 1.95 μCi of ^3^ H]TTP (120 Ci/mmol; MP Biomedicals Inc., Irvine, CA. USA) [34]. Samples were preincubated for 5 min at 37°C. The reaction was started by the addition of 12 nM enzyme. After 5 min, 7.5 μl aliquots were taken out and spotted on DEAE filter paper. Filters were washed twice with 2 x SSC (300 mM NaCl, 30 mM Na-citrate, pH 7.0) for 10 minutes, twice with 96 % ethanol for 10 min and dried. Incorporation of radiolabeled precursor into polydeoxynucleotide was determined by liquid scintillation counting. Under the conditions used 1 unit (U) of enzyme catalyzes the incorporation of 1 nmol TTP into poly(rA)/oligo(dT)_15_ in 10 min at 37°C.

### Structure alignments

Structure alignments shown in Figure 5 were performed using the program WinCoot [35].

### Accession codes

The structure coordinates of the wt PFV RNase H-(Q^591^-N^751^) domain were deposited in the Protein Data Bank under the accession code 2LSN. Chemical shift assignments were deposited in the BioMagResBank, accession number: 17745 [5].

## Abbreviations

B. halodurans, Bacillus halodurans ; DTT, Dithiothreitol; E. coli, Escherichia coli ; FV, Foamy virus; GB1, Immunoglobulin binding domain B1 of streptococcal protein G; HIV-1, Human immunodeficiency virus type 1; PFV, Prototype foamy virus; MoMLV, Moloney murine leukemia virus; PPT, Polypurine tract; PR, Protease; RHBD, RNA-hybrid binding domain; RNase H, Ribonuclease H; RT, Reverse transcriptase; SFVmac, Simian foamy virus from macaques; TEV, Tobacco etch virus; XMRV, Xenotropic murine leukemia virus-related virus.

## Competing interests

The authors declare that they have no competing interests.

## Authors' contributions

BMW conceived and coordinated the study. BL performed the experiments. BL, BMW, MJH and KS analyzed the data. BMW, BL and KS wrote the paper. PR provided conceptual input. All authors read and approved the manuscript.
